# Dietary inflammatory index and unfavorable dietary patterns associated with ischemic stroke in China

**DOI:** 10.6133/apjcn.202604_35(2).0009

**Published:** 2026-03-17

**Authors:** Qunli Xu, Qianyi Chen, Yiyu Zhuang, Lanlan Zhou, Lanjun Shen, Tingting Li, Zhefang Hu, Ronghua Zhang, Danting Su, Lijun Feng

**Affiliations:** 1 Nursing Department, Sir Run Run Shaw Hospital, Zhejiang University School of Medicine, Hangzhou, China; 2 Department of Clinical Nutrition, Sir Run Run Shaw Hospital, Zhejiang University, Hangzhou, China; 3 Zhejiang Provincial Center for Disease Control and Prevention, Hangzhou, China; † Both authors contributed equally to this manuscript

**Keywords:** ischemic stroke, dietary patterns, dietary inflammatory index, risk, case-control study

## Abstract

**Background and Objectives:**

The dietary inflammatory potential, assessed by the Dietary Inflammatory Index (DII), may influence ischemic stroke (IS) risk, but evidence from high-incidence regions in China remains limited. This study aimed to investigate the associations among dietary patterns, the DII, and IS in Eastern China.

**Methods and Study Design:**

In a hospital-based case-control study in Zhejiang, China, 223 IS patients and 510 age- and sex-matched controls completed a validated food frequency questionnaire. DII scores were calculated, and dietary patterns were derived using factor analysis. Multivariate logistic regression models were used to calculate odds ratios and 95% confidence intervals (CIs).

**Results:**

A “Jiangnan red-sauce and heavy oil” pattern, characterized by high intake of refined grains, salted vegetables, processed meats, and fats, was associated with higher DII scores and an increased IS risk (OR = 1.85; 95% CI: 1.74–2.51; top versus bottom tertile). Conversely, a “Traditional Chinese” pattern, rich in whole grains, vegetables, fruits, and legumes, was correlated with lower DII scores and a potentially reduced IS risk (OR = 0.85; 95% CI: 0.76–0.94).

**Conclusions:**

The findings suggest that pro-inflammatory diets were associated with a high likelihood of IS, while anti-inflammatory patterns, such as the Traditional Chinese diet, may be protective. The findings may also provide insights for dietary prevention strategies in the high-risk populations, pending confirmation from prospective studies.

## INTRODUCTION

Ischemic stroke (IS) is one of the most common types of stroke, typically resulting from a blockage in an artery or, rarely, a vein, accounting for approximately 87% of all strokes.^1, 2^ According to the World Health Organization, stroke and other cerebrovascular diseases are the second leading causes of death globally,^3^ responsible for 9.7% of total mortality. In China, the prevalence of stroke has increased significantly over the past three decades, becoming the leading cause of death among residents and presenting major challenges for prevention and control.^1, 3^ In 2019, China recorded 28.8 million stroke patients, including 3.94 million new cases and 21,900 stroke-related deaths.^4^ Over the last 30 years, rapid health transitions and socio-demographic changes in China have contributed to shifting disease patterns.^5, 6^ The prevalence of hypertension,^7^ smoking,^8^ obesity, 1and diabetes^9^ has risen significantly, all of which are associated with the increasing incidence of stroke.^10, 11^

Although the exact causes and mechanisms of stroke remain incompletely understood, inflammation is considered to play a central role in its onset and progression.^12,13^ Inflammation is a natural protective response to injury or infection, but sustained, chronic inflammation can lead to endothelial dysfunction, platelet activation, and a pro-thrombotic state, which increases the risk of stroke.^14^

Diet is a crucial factor influencing chronic inflammation levels in the human body.^15^ The classic Western diet is recognized as pro-inflammatory,^16,17^ while Mediterranean and Oriental diets are considered anti-inflammatory and are associated with reduced risks of cardiovascular diseases in the general population.^18, 19^ Certain nutritional components and individual foods, such as vegetables and fruits, have been shown to lower chronic inflammation levels. The Dietary Inflammatory Index (DII) is a numerical measure that evaluates the inflammatory potential of a person’s diet.^20^ Introduced in 2009^21^ and refined by Shivappa et al. in 2013,^22^ DII has been developed and validated as an index for assessing diet-related systemic inflammation. It is based on the effects of 45 foods on inflammatory biomarkers, including C-reactive protein (CRP) and interleukin-6 (IL-6).^23^ Individual food DII scores are summed to calculate an overall DII, which ranges theoretically from −8.87 (highly anti-inflammatory) to +7.89 (highly pro-inflammatory).^24^

Currently, treatment options for stroke remain limited.^25^ Understanding the risk factors and implementing preventive measures are critical to reducing stroke incidence. Although the relationship between DII, stroke, and dietary patterns is complex and subtle, clear evidence linking these factors is lacking. This study aimed to investigate the associations among dietary patterns, DII, and IS in adults from Zhejiang Province, Eastern China. Given that higher DII scores have been associated with elevated inflammatory biomarkers and increased cardiometabolic risk in previous studies,^26, 27^ we hypothesized that pro-inflammatory dietary patterns would be associated with higher DII scores and with greater risk of IS, whereas anti-inflammatory dietary patterns would show the opposite direction. In this study, the DII was used as an indicator of the inflammatory potential inherent in each dietary pattern rather than a mediator. This region, chosen as the study site because it combines relatively high level of economic development with a notable burden of IS, making it a suitable setting for exploring the determinants of stroke risk.

## METHODS

### Subjects and study design

This was a hospital-based matched case-control study conducted between February 2023 and February 2024 at Sir Run Run Shaw Hospital in Hangzhou, Zhejiang Province, China. A total of 223 participants newly diagnosed with IS were enrolled, and 510 age- and sex-matched controls were recruited. Inclusion criteria were: (a) age between 18 -70 years; (b) diagnosis of IS confirmed by computed tomography or magnetic resonance imaging in the outpatient clinic;(c) diagnosis within the previous three months to ensure data reliability. Exclusion criteria included: (a) body mass index (BMI) <19 kg/m², (b) heavy cigarette smoking (>20 cigarettes/day), (c) heavy alcohol consumption (>1000 mL of beer or equivalent drinks/day), (d) heavy consumption of caffeinated beverages (>5 cups of brewed tea or equivalent drinks/day); (e) severe hypertension (Systolic blood ≥160 mmHg or Diastolic blood pressure ≥100 mmHg or on intensive antihypertensive treatment); (f) poorly controlled diabetes (Fasting plasma glucose ≥11.1 mmol/ L,Glycated hemoglobin A1c ≥9%, or insulin-treated patients); (g) severe dyslipidemia (Triglycerides ≥5.6 mmol/L or Low-density lipoprotein ≥4.9 mmol/L or on intensive lipid-lowering therapy); (h) incomplete or inconsistent data.

**Figure 1:**
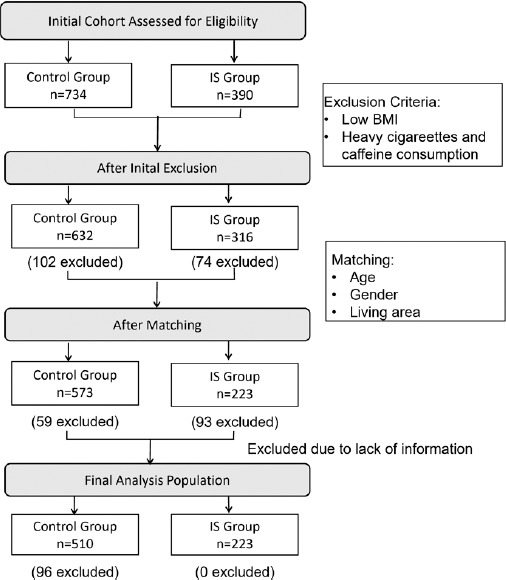
Enrollment and Matching Reasons for participant exclusion included low body mass index (BMI <18.5 kg/m²) or high BMI (≥40 kg/m²), heavy cigarette smoking (>20 cigarettes/day), heavy alcohol consumption (>1000 mL of beer or equivalent drinks/day), heavy caffeine intake (>5 cups of brewed tea or equivalent drinks/day), unmatched epidemiological characteristics, and loss to follow-up. IS indicates ischemic stroke.

Controls were recruited from the same hospital and nearby communities during the same period as the cases. They included individuals undergoing routine health examinations who had no history of stroke, cardionvascular disease, or other neurological disorders. Controls were frequency-matched to cases by age (±2 years), sex, and residential area to ensure comparability. The study was initially designed to achieve an approximate 1:2 case-to-control ratio. Due to exclusions and incomplete data, the final sample available for analysis comprised 223 IS cases and 510 controls. Inclusion criteria: stable vital signs, no history of stroke, neurological disease (e.g., Parkinson’s disease, dementia, or paralysis), or antidepressant use (Figure 1). All participants provided informed consent prior to participation. To minimize recall bias, only patients whose IS diagnosis occurred within the previous 3 months were included. Dietary data were collected after clinical stabilization but before any dietary counseling.

### Human ethics and consent to participate

The study was approved by the Ethics Committee of Sir Run Run Shaw Hospital, Zhejiang University School of Medicine (No. 0503, 2024). All participants were informed about the study protocol and provided written informed consent to participate in the study. All methods and procedures were carried out in accordance with the relevant guidelines and regulations, and with the ethical standards laid down in the 1964 Declaration of Helsinki and its later amendments.

### General and anthropometric measurements

All medical tests were conducted on the same day. Trained nurses collected data on participants’ demographic characteristics, medical history, lifestyle, and anthropometric parameters. In addition to excluding severe cases, the following clinically diagnosed medical histories were collected and used as binary covariates in multivariate models: hypertension, hyperlipidemia, and diabetes mellitus. Body weight and height were measured using standardized equipment, and BMI was calculated as weight (kg) divided by height squared (m²). Blood pressure (BP) was measured twice in the seated position after a 10-minute rest using a calibrated sphygmomanometer.

### Assessment of dietary intake

Dietary intake during the 12 months preceding disease onset was assessed using a 143-item semi-quantitative food frequency questionnaire (FFQ) adapted from the 2020 China National Nutrition and Health Survey (CNNHS). The FFQ covered nine food groups and 20 categories, including rice and rice products; other cereals and their products; vegetables; fresh fruits; red meat; poultry; fish and seafood; eggs; dairy products; soy products; fried foods; pickled food and nuts; packaged snacks; vegetable oil/lard; condiments; carbonated beverages; fruit juices and plain water (250 mL/cup). The FFQ was interviewer-administered by trained nutritionists through face-to-face interviews. Standardized food photographs and portion-size charts were used to assist participants in estimating typical consumption. When patients could not recall accurately, a family member familiar with the participant’s diet assisted the interview. This FFQ has been previously validated for Chinese adults. Reported intake frequencies and portion sizes were converted to grams/day for analysis.

### The DII assessment

The DII developed by researchers at the University of South Carolina as part of a cancer prevention and control initiative, was used to measure the inflammatory potential of participants’ diets.^20^ The DII calculation linked individual dietary data obtained from the FFQ with global average intake levels to compute Z-scores:

Z-score = (Individual’s daily intake of dietary component - Global average intake of dietary component) / (Standard deviation of global intake × Inflammatory effect score of each dietary component)

The Z-scores were converted to a percentile scale, doubled, and adjusted by subtracting “1” to create a symmetric distribution centered around “0.” These adjusted values were then multiplied by the inflammatory score for each dietary component. The sum of these values yielded the individual’s DII score, expressed as: Total DII =∑(ni × xi × bi).

In this study, the DII was calculated using 14 out of the 45 standard dietary components, based on data obtained from the FFQ. These components—energy, protein, fat, carbohydrates, dietary fiber, cholesterol, vitamin A, vitamin B1, vitamin B2, vitamin C, vitamin E, calcium, iron, and zinc—were selected due to their availability and reliability in the validated Chinese FFQ. Although this reduced set may not fully capture the overall inflammatory potential of the diet, previous research has demonstrated that partial DII calculations can still provide meaningful and valid associations with inflammation-related health outcomes.^28^

### Measurements of dietary patterns and statistical analyses

Data were analyzed using SPSS 17.0 software (SPSS Inc., Chicago, IL, USA). Continuous variables were expressed as means ± standard deviations (SDs) or medians (interquartile ranges), and categorical variables as counts and percentages. Differences between cases and controls were compared using independent-samples t-tests or chi-square tests, as appropriate. Variables with *p* <0.05 in univariate analyses were tested for multicollinearity using variance inflation factors (VIFs). A VIF < 10 was considered to indicate no multicollinearity. Variables without multicollinearity were then entered into the multivariate logistic regression model to identify independent factors associated with IS. As frequency matching was used, unconditional multivariate logistic regression was employed to estimate associations, adjusting for the matching variables (age, sex, and residential area) and other covariates. Logistic regression models were constructed to evaluate odds ratios (ORs) and 95% confidence intervals (CIs) for associations among the dietary patterns, DII scores, and IS. Model 1 was unadjusted. Model 2 adjusted for age, sex, BMI, marital status, insurance status (basic medical insurance for urban workers, urban resident basic medical insurance, or new rural cooperative medical scheme), smoking status (current smoker or non-smoker), alcohol consumption (current drinker or non-drinker), hypertension, and diabetes; Model 3 further adjusted for total energy and percentage of energy from fat.

Exploratory factor analysis was applied to identify dietary patterns. From 100 food items, 24 were selected for analysis using principal component analysis. When considering the retained factors, we evaluated the eigenvalue (≥1.5), scree plot, factor interpretability and variance explained (> 5%) to determine which set of factors best described the different dietary patterns. Items remained in a factor if they had an absolute value ≥|0.40| with that pattern. The cumulative variance of the retained factors explained the variation in food intake. Standard regression factor scores were calculated for each participant and dietary pattern representing their level of adherence to that pattern. For analysis, these continuous factor scores were then categorized into tertiles (T1-T3), with T1 representing the lowest adherence to that pattern and T3 the highest adherence. A significance level of 0.05 was used for hypothesis testing.

## RESULTS

### Study population demographics

The baseline characteristics of IS patients and matched controls are presented in Table 1. The groups were comparable in terms of age, both as a continuous variable (64.8 ± 12.2 years for cases vs. 63.5 ± 11.1 years for controls, p = 0.176) and across age strata (*p* = 0.117), based on the 2002 Chinese National Health Survey standard. However, significant differences were observed in several key variables. Compared with the control group, stroke participants exhibited higher proportions of males, BMI, current alcohol consumption,with underlying conditions (diabetes mellitus, hypertension and hyperlipidemia) (*p* <0.05). No significant differences were found between the groups in terms of marital status, residence area, and smoking behavior. Analysis of FFQ data revealed that IS patients had significantly higher daily intakes of total energy (2140 ± 320 kcal vs. 1950 ± 220 kcal, *p* < 0.001) and fat (87.8 ± 11.5 g vs. 73.4 ± 10.5 g, *p* < 0.001), as well as a higher percentage of energy from fat (37.0 ± 0.9% vs. 33.9 ± 3.2%, *p* < 0.001). No significant differences were detected in the intake of protein or carbohydrates.

### Analysis of DII in all subjects based on the FFQ

In this study, the DII scores were predominantly positive, with an average score of 1.69 (95% CI: -2.46 to 2.41), indicating that the overall diet was pro-inflammatory. The DII scores were divided into three tertiles: T1 (-2.37 to 1.18), T2 (1.18 to 1.85), and T3 (1.85 to 2.50). The T1 group represented the most anti-inflammatory diet, while the T3 group represented the most pro-inflammatory diet. Except for rice and rice products, differences in dietary intake amounts and other dietary comparisons across the DII groups were statistically significant (*p* <0.001) (Table 2).

### Dietary patterns and characteristics

Factor analysis extracted three main factors with eigenvalues of 19.79, 10.03, and 5.60, respectively, explaining 35.4% of the total variance. Each dietary pattern was named according to the food group with the highest absolute factor loading. Three major dietary patterns were identified by factor analysis: the unique culinary style of the ‘Jiangnan diet-red sauce and heavy oil’ in the Chinese Jiangnan region, which is characterized by a high frequency of high intakes of refined grains and their products, pickled vegetables, salted-cured meat, and cooking oils or lard; the ‘Traditional Chinese’ pattern, which is characterized by high intakes of whole grains and related products, vegetables and fruit, beans and their products; the ‘High-fat and high-meat’ dietary pattern, charactered by high intakes of raw meat, aquatic products, and milk and its products. The factor loading matrices for these dietary patterns are shown in Table 3.

### Association between dietary pattern and DII

Before conducting the multivariate logistic regression, potential multicollinearity among all covariates was examined using VIFs. No significant collinearity was detected (all VIFs < 3); therefore, age, sex, smoking, alcohol intake, BMI, total energy intake, percentage energy from fat, hypertension, diabetes were retained in the final model. To validate the inflammatory potential inherent in the identified dietary patterns, we cross-tabulated the adherence to each pattern (expressed in tertiles) with the DII scores. The results, presented in Table 4, demonstrate that the dietary pattern we identified were strongly associated with their expected inflammatory profiles, as quantified by the DII.

As hypothesized, higher adherence to the pro-inflammatory ‘Jiangnan diet red-sauce and heavy oil’ pattern was significantly associated with higher DII scores. Participants in the highest tertile (T3) of adherence to this pattern had a significantly higher likelihood of also being in a higher DII category compared with those in the lowest tertile (T1), with a fully adjusted OR of 1.85 (95% CI: 1.74–2.51). This finding confirms that this pattern is characterized by strong pro-inflammatory pro-inflammatory dietary properties.Conversely, and also as expected, higher adherence to the anti-inflammatory ‘Traditional Chinese’ pattern was associated with lower DII scores. Participants in the highest tertile (T3) of adherence to this pattern had a lower likelihood of being in a high DII category (adjusted OR = 0.85; 95% CI: 0.76–0.94). It is important to note that this OR below 1 does not imply that a high DII is protective; rather, it indicates that individuals who closely follow the ‘Traditional Chinese’ pattern are less likely to consume a diet with high inflammatory potential. This pattern is intrinsically associated with an anti-inflammatory dietary profile.

No significant association was observed between the ‘High-fat and high-meat’ pattern and DII scores, suggesting that its inflammatory impact may be relatively neutral or influenced by unmeasured dietary factors not captured by the DII in this study.

## DISCUSSION

In this case-control study, 733 participants were recruited to assess the correlation between the DII, dietary patterns, and the risk of IS. Conducted in Zhejiang Province, Eastern China, this study observed that varying degrees of dietary imbalance were associated with IS risk in a Chinese adult population. Participants with higher adherence to the unfavorable ‘Jiangnan red-sauce and heavy oil’ dietary pattern, and lower adherence to the ‘Traditional Chinese’ pattern, exhibited higher DII scores. Although causality cannot be inferred from the present design, these findings suggest that dietary patterns with higher inflammatory potential may contribute to stroke occurrence or reflect underlying lifestyle risk factors in populations undergoing rapid nutritional transitions.

**Table 1:** Baseline characteristic of IS cases and controls

Characteristic	IS (Case group) (N=223)	Non-IS (Control group) (N=510)	*χ*^2^/*t*	*p*-value
Gender			4.36	0.037
Males	141 (63.2)	216 (45.3)		
Females	82 (36.8)	293 (54.7)		
Age (years)	64.8 ± 12.2	63.5 ± 11.1	1.42	0.176
Age (years), n (%)			6.50	0.117
≤45	44 (19.7)	105 (20.6)		
46~	41 (18.4)	71 (13.9)		
56~	64 (28.7)	127 (24.9)		
66~	57 (25.6)	82 (16.1)		
≥76	17 (7.6)	125 (24.5)		
BMI (kg/m^2^)	23.6 ± 2.9	21.6 ± 2.8	8.71	<0.0001
Marital status, n (%)			2.48	0.115
Married	187 (83.9)	409 (80.2)		
Unmarried	36 (16.2)	101 (13.0)		
Insurance state^†^			3.15	0.207
Basic medical insurance for urban workers	77 (34.52)	167 (32.8)		
Urban resident basic medical insurance	112 (50.2)	267 (52.3)		
New rural cooperative medical scheme	34 (15.2)	76 (14.9)		
Cigarette smoking^‡^, n (%)			1.90	0.191
Current smokers	30 (13.5)	84 (16.5)		
Non-smokers	193 (86.5)	426 (83.5)		
Alcohol intake^‡^, n (%)			5.07	0.024
Current drinkers	69 (30.9)	122 (23.9)		
Non-drinkers	153 (69.1)	388 (76.1)		
Diabetes, n (%)			99.86	<0.001
No	142 (63.7)	431 (84.5)		
Yes	81 (36.3)	79 (15.5)		
Hypertension, n (%)			216.97	<0.001
No	52 (23.4)	328 (64.4)		
Yes	171 (76.6)	182 (35.6)		
Hyperlipidemia, n (%)			59.31	<0.001
No	24 (10.6)	157 (30.7)		
Yes	199 (89.4)	353 (69.3)		
Nutrients intake				
Energy (kcal)	2,140 ± 320	1,950 ± 216	15.8	<0.001
Protein (g)	79.0 ± 11.8	76.5 ± 11.5	1.67	0.164
% Total energy	14.8 ± 0.9	14.9 ± 1.6	1.74	0.657
Carbohydrate (g)	259 ± 43.7	250± 35.8	2.41	0.111
% Total energy	48.2 ± 1.5	47.1 ± 4.4	1.83	0.524
Fats (g)	87.8 ± 11.5	73.4 ± 10.5	11.0	<0.001
% Total energy	37.0 ± 0.9	33.9 ± 3.2	4.11	<0.001

† Usually refers to the need for continuous insurance coverage in the participating area for 6 months or more.

‡ Smoking 1 or less than 20 cigarettes per day for a period of 6 months or more consuming alcohol at least once per day, or ≤the equivalent of 5 beers per day.

Dietary patterns—particularly those involving higher intakes of beneficial foods, nutrients-dense—may modulate inflammatory cytokines expression and serve as a promising approach to manage cardiovascular risk factors.^29, 30^ Our results further revealed that adherence to the ‘Jiangnan’ diet was significantly associated with an increased risk of IS. A significant positive association was observed between higher dietary inflammatory potential and increased IS risk. Participants in the highest DII tertile (T3), representing the most pro-inflammatory diets, had a significantly greater likelihood of IS compared with those in the lowest tertile (T1), with an adjusted OR of 1.85 (95% CI: 1.74–2.51). These findings are consistent with the hypothesis that pro-inflammatory diets may be linked to the pathogenesis of IS and further suggest the DII could serve as a valuable tool for identifying high-risk dietary profiles. Our result are line with a nationwide prospective cohort study in China, which reported that lower adherence to healthy, sustainable, plant-based dietary patterns were associated with higher DII scores (Q5 vs. Q1: HR = 1.90; 95% CI: 1.26–2.88; *p*-trend = 0.0006).^31^ The ongoing nutritional transition in China—marked by rising fat intake, increased availability of processed foods, and declining consumption of whole grains—has been well documented.^32, 33^ Zhejiang, as one of the most economically developed regions, has experienced particularly rapid dietary modernization. In this context, the ‘Jiangnan red-sauce and heavy oil’ pattern identified in our factor analysis likely represents a modernized variant of the traditional Jiangnan diet.^32^ The shift toward prolonged braising, heavier use of soy sauce, and more frequent consumption of animal fats may have increased the intake of sodium, saturated fat, and total energy.^34^ At the same time, reduced consumption of coarse grains and legumes may have contributed to lower dietary fiber intake, which is consistent with higher dietary inflammatory potential as assessed by the DII—a measure validated against circulating inflammatory biomarkers.^27^ On the other hand, adherence to the ‘Traditional Chinese’ diet, was associated with a lower OR for IS. In the highest tertile (T3) of the DII, the adjusted OR for IS was 0.85 (95% CI: 0.76–0.94). Similar patterns have been observed with the Mediterranean diet, which features a low inflammatory potential and higher intakes of fruits, vegetables, whole grains, and unsaturated fatty acids. This dietary pattern has been shown to reduce circulating inflammatory cytokine levels, attenuate atherosclerosis, and lower the risk of cardiovascular mortality.^35-37^ Therefore, the observed associations with IS in our population may reflect the synergistic effects of multiple dietary components inherent in this modernized pattern rather than the influence of any single food item.^38, 39^

**Table 2:** Comparison of food intake across different DII groups in the study population. [M (P25, P75), n=733]

Food group	T1 (n=244)	T2 (n=245)	T3 (n=244)	*p-*value^†^
Rice and rice products	231 (148, 261.9)	250 (227, 283)	220 (143, 334)	0.187
Other cereals and products (millet, buckwheat) (g/day)	75.0 (33.0, 150)	49.5 (16.5, 80.6)	15.8 (10.7, 33.0)	<0.001
Fresh vegetables (g/day)	382 (200, 511)	316.5 (195, 375)	251 (116, 380)	<0.001
Fresh fruits (g/day)	119 (75.0, 150)	49.5 (16.5, 75.0)	10.5 (4.5, 33.0)	<0.001
Red meat (g/day)	50.6 (29.0, 122)	49.5 (31.3, 82.5)	20.6 (9.8, 55.0)	<0.001
Poultry (g/day)	69.8 (27.0, 111)	49.5 (32.3, 85.5)	21.8 (9.0, 54.0)	<0.001
Animal organs (liver, kidneys, stomach, tongue, heart, intestines)	10.0 (4.50, 28.0)	9.0 (4.50, 15.0)	6.80 (2.30, 12.8)	0.003
Fish, shrimp, and seafood (g/day)	10.5 (4.5, 22.1)	9.0 (4.5, 15.0)	6.8 (2.3, 12.8)	0.005
Fresh eggs (chicken eggs, duck eggs, goose eggs, quail eggs) (g/day)	37.5 (16.5, 75.0)	18.8 (5.30, 37.5)	9.0 (2.30, 27.7)	<0.001
Milk and dairy products (g/day)	87.0 (16.9, 300)	66.0 (9.0, 150)	16.5 (4.5, 49.5)	<0.001
Soy products (g/day)	49.5 (25.1, 79.5)	35.3 (16.5, 51.5)	12.0 (5.3, 27.0)	<0.001
Fried foods (g/day)	20.0 (10.0, 35.0)	30.0 (15.0, 75.0)	40.0 (20.0, 90.0)	<0.001
Pickled vegetables (g/day)	37.8 (15.0, 60.0)	50.0 (25.0, 75.0)	30.0 (15.0, 50.0)	0.018
Nuts (peanuts, sunflower seeds) (g/day)	50.0 (25.0, 75.0)	30.0 (15.0, 50.0)	10.0 (5.0, 25.0)	<0.001
Packaged snacks (g/day)	25.0 (10.0, 40.0)	35.0 (15.0, 55.0)	45.0 (20.0, 70.0)	<0.001
Vegetable oil (g/day)	20.0 (10.0, 35.0)	30.0 (15.0, 50.0)	40.0 (20.0, 65.0)	<0.001
Salt	5.0 (2.5, 8.0)	7.5 (4.0,11.0)	10.0 (5.0, 15.0)	<0.001
Carbonated beverages & fruit juice drinks (mL/day)	100 (50.0, 150)	150.0 (75.0, 225)	200.0 (100,300)	<0.001
Wine (mL/day)	50.0 (25.0, 75.0)	75.0 (37.5, 113)	100.0 (50.0, 150)	<0.05

† *p* represents the results of the Kruskal-Wallis rank sum test for inter-group difference analysis.

**Table 3:** Rotated factor loading matrix for the three dietary patterns identified by factor analysis among 733 subjects^†^

Food groups	Dietary patterns		
	Jiangnan diet-red sauce and heavy oil	Traditional Chinese	High-fat and high-meat
Refined grains and their products	0.581	-	-
Whole grain and related products	-	0.764	-
Vegetables and fruits	-	0.650	-
Raw meats	-	-	0.561
Pickled vegetables (fermented or salt)	0.510	-	-
Salted-cured meats	0.743	-	0.640
vegetable oil/ lard	0.560	-	-
Aquatic products	-	-	0.757
Milk and its product	-	-	0.563
Beans and their products	-	0.576	-

† The food items were considered to be strongly associated with the dietary pattern that factor load absolute values were more than 0.4

Previous studies have primarily examined the association between the DII and Cardiovascular Disease (CVD). In the RaNCD cohort study,^40^ which included 9% of participants with prior CVD records, researchers investigated the association between DII and CVD risk. The findings revealed that greater adherence to a pro-inflammatory diet was associated with an increased risk of CVD (OR: 1.40, 95% CI: 1.10–1.80). These findings lend additional support to our observation that pro-inflammatory dietary patterns are associated with an elevated risk of IS. Chronic inflammation plays a pivotal role in the development of atherosclerosis, the primary pathological process underlying most cases of IS. Pro-inflammatory diets, such as the ‘Jiangnan’ diet, have been reported to be associated with elevated circulating inflammatory biomarkers, including CRP, tumor necrosis factor-α, and interleukin-6 (IL-6).^41-43^ These biomarkers are believed to play a role in endothelial dysfunction, plaque formation, and thrombosis.^44^ Additionally, emerging evidence suggests that pro-inflammatory diets can alter the gut microbiome, increasing intestinal permeability and enabling bacterial endotoxins to enter the bloodstream, which triggers systemic inflammation.^45^ Eckburg PB et al. reported that stroke patients often exhibit an enriched presence of intestinal *Enterobacteriaceae* and reduced *Clostridiaceae* and *Lachnospira*, potentially linked to elevated inflammatory responses or infections following stroke.^46^ These alterations are associated with severe brain injury and poorer stroke outcomes.^47^ While this study primarily focused on assessing the inflammatory potential of diets through DII and its association with IS risk, these findings underscore the need for future research into the interactions between dietary patterns, gut microbiota, and IS risk. This could provide valuable insights into the underlying mechanisms and inform novel preventive strategies.

**Table 4:** Odd Ratios (ORs) and 95% Confidence Intervals (CIs) for having DII scores according to tertiles of adherence to each dietary pattern^†^

Dietary pattern	Tertiles of pattern adherence	OR *(95% CI)* for high DII score^†^		
		Model 1^‡^ OR (*95%CI*)	Model 2^§^ OR (*95%CI*)	Model 3^¶^ OR (*95%CI*)
Jiangnan diet-red sauce and heavy oil	T1	1	1	1
	T2	1.11 (0.89, 1.73)	1.11 (0.41, 1.85)	1.08 (0.41, 1.92)
	T3	1.24 (1.21, 1.49)*	2.04(1.96, 2.54)*	1.85 (1.74, 2.51)*
Traditional Chinese	T1	1	1	1
	T2	0.90 (0.81, 1.00)	0.92 (0.83, 1.02)	0.92 (0.83, 1.02)
	T3	0.84 (0.75, 0.94)**	0.84(0.76, 0.94)**	0.85 (0.76, 0.94)**
High-fat and high-meat	T1	1	1	1
	T2	0.98(0.67,1.09)	0.99 (0.89, 1.10)	0.99 (0.89, 1.10)
	T3	0.93 (0.84, 1.04)	0.94 (0.84, 1.05)	0.93 (0.84, 1.04)

OR, odd ratio; CI: confidence interval

† Dietary Inflammatory Index tertile ranges: T1 (-2.37 to 1.18), T2 (1.18 to 1.85), T3 (1.85 to 2.50)

‡ Model 1: unadjusted

§ Model 2: adjusted for age, marital status, BMI, insurance status, smoking, alcohol consumption, hypertension, hyperlipidemia, and diabetes melltius

¶ Model 3: further adjusted for total energy and % energy from fat

* *p* <0.05;

** *p* <0.01

The current study possesses several notable strengths. First, it is among the few investigations conducted in a Chinese population to explore the associations between the DII and empirically derived dietary patterns, capturing both pro-inflammatory and anti-inflammatory dietary profiles. Second, from a public-health perspective, our findings highlight the importance of distinguishing traditional regional dietary cultures from their contemporary variants. Although the traditional Jiangnan cuisine has long been regarded as health-promoting, the contemporary dietary pattern prevalent in urban Zhejiang may have diverge substantially from its historical roots.

Nevertheless, several limitations of this study should also be acknowledged. First, the DII was calculated using only 14 dietary components due to limitations in the FFQ. These components may have reduced the index’s ability to fully capture the inflammatory potential of the overall diet and introduced the potential exposure misclassification, Second, although the three dietary patterns identified in the study are interpretable and biologically plausible, the cumulative variance explained by these patterns was 35.4%. This suggests that a considerable proportion of the variation in overall dietary behavior was not captured by these patterns—a common methodological limitation in dietary pattern research. Third, the FFQ required participants to recall their dietary intake approximately one year prior to disease onset. Such long-term retrospective recall is particularly challenging for stroke patients and may have increased the risk of differential recall bias, while trained interviewers and standardized procedures were employed to minimize such bias, differential misreporting between groups cannot be entirely excluded. Finally, because of the observational nature of the case-control design, causal inferences cannot be established. These limitations underscore the need for large-scale prospective cohort studies with more detailed dietary data to confirm and extend our findings. Despite these limitations, the present study provides valuable insights into the role of dietary inflammation in the etiology of IS and offers an important foundation for future research and public health initiatives aimed at diet-related stroke prevention among high-risk populations.

### Conclusion

In conclusion, this case-control study found that greater adherence to the unfavorable ‘Jiangnan red-sauce and heavy oil’ dietary pattern and lower adherence to the ‘Traditional Chinese’ dietary pattern were associated with higher DII scores in this sample. Our findings suggest that a pro-inflammatory diet, as indicated by higher DII scores, was associated with higher odds of having IS in Chinese adults. There findings are consistent with the notion that adherence to pro-inflammatory dietary habits may adversely affect cardiometabolic health, whereas adherence to anti-inflammatory dietary pattern with lower DII scores may be linked to a reduced risk and burden of IS. Further large-scale prospective studies are warranted to establish precise associations between dietary variables and the occurrence of IS.

## DISCLOSURE ON THE USE OF AI AND AI-ASSISTED TECHNOLOGIES

All authors declare that no AI or AI-assisted technologies were used in the data collection, analysis, or creation of images and graphical elements for this study. The authors reviewed and edited the content and takes full responsibility for the content of the publication.

## CONFLICT OF INTEREST AND FUNDING DISCLOSURES

The authors declare no conflict of interest.

The study was supported by The General Research Program of Medical and Hygiene from the Health and Family Planning Commission in Zhejiang Province (2024KY1144) and The National Health Commission Research Fund of China (WKJ-ZJ-2304). The funders had no role in study design, data collection, and analysis, decision to publish, or preparation of the manuscript.
